# MammaPrint versus EndoPredict: Poor correlation in disease recurrence risk classification of hormone receptor positive breast cancer

**DOI:** 10.1371/journal.pone.0183458

**Published:** 2017-08-29

**Authors:** Andreas Bösl, Andreas Spitzmüller, Zerina Jasarevic, Stefanie Rauch, Silke Jäger, Felix Offner

**Affiliations:** 1 Department of Molecular Diagnostics, Institute of Pathology, Academic Teaching Hospital Feldkirch, Feldkirch, Austria; 2 Department of Genome Oriented Bioinformatics, Technical University of Munich, Freising, Germany; 3 Department of Surgery, Academic Teaching Hospital Feldkirch, Feldkirch, Austria; 4 FH Campus Vienna, University of Applied Sciences, Vienna, Austria; University of North Carolina at Chapel Hill School of Medicine, UNITED STATES

## Abstract

**Introduction:**

Correct risk assessment of disease recurrence in patients with early breast cancer is critically important to detect patients who may be spared adjuvant chemotherapy. In clinical practice this is increasingly done based on the results of gene expression assays. In the present study we compared the concordance of the 70-gene signature MammaPrint (MP) with the 12 gene assay EndoPredict (EP).

**Methods:**

Representative tissue of 48 primary tumours was analysed with the MP during routine diagnostic purposes. Corresponding formalin-fixed, paraffin-embedded tissue was thereafter analysed by the EP test. Risk categories of both tests were compared.

**Results:**

41 of 48 tumours could be directly compared by both tests. Of the 17 MP low risk cases, only 9 were considered low risk by EP (53% agreement) and of the 24 MP high risk cases, 18 were high risk by EP (75% agreement). Discrepancies occurred in 14 of 41 cases (34.1%). There was only a weak and non-significant correlation between the MP and EP test with an overall concordance of only 66%. The original therapeutic recommendation was based on the MP and would have been changed in 38% of the patients following EP test results. 4 patients developed distant metastases. The respective tumours of these patients were all classified as high risk by the EP, but only 3 were classified as high risk by the MP.

**Conclusion:**

Both tests resulted in different treatment recommendations for a significant proportion of patients and cannot be used interchangeably. The results underscore the urgent need for further comparative analyses of multi-genomic tests to avoid misclassification of disease recurrence risk in breast cancer patients.

## Introduction

Every year approximately one and a half million new cases of breast cancer are registered worldwide [1]. While breast cancer mortality has substantially dropped over the past decades due to earlier detection and adjuvant therapies it is still difficult to tailor adjuvant chemotherapy for individual patients. The risk of recurrence and indication to administer chemotherapy is largely dependent on the classical clinic-pathological prognostic factors of tumour size, involvement of lymph nodes, histologic grade, expression of hormone receptors, and human epidermal growth factor receptor 2 (HER2) amplification [2,3,4,5]. While these factors aid in the determination of disease recurrence risk it remains a particular problem to identify those patients unlikely to benefit from adjuvant chemotherapy. It has been estimated that in early-stage breast cancer only 15% of tumours will recur suggesting that based on current treatment guidelines many patients will receive chemotherapy without benefit [2,3,4,6]. Given this dilemma there is great interest in the diagnostic application of prognostic and predictive multi-gene expression assays. In clinical practice, these multi-gene classifiers are currently mostly used for estrogen (ER)-positive/HER2-negative breast cancer. The tests primarily rely on quantification of ER- and proliferation-related genes and combine these into multivariate prediction models.

MammaPrint is one of the first-generation prognostic signatures. It is a micro-array based 70 gene assay, which was launched in 2002 [7]. In validation studies the MammaPrint assay was shown to successfully predict metastasis free survival and overall survival and it has been suggested that it might be useful to select patients not requiring adjuvant chemotherapy [7,8,9]. The most recently published results of the large prospective study of the MammaPrint assay (MINDACT) showed that approximately 46% of women with early-stage breast cancer with a high clinico-pathological risk and low genomic risk might not require chemotherapy [10].

EndoPredict is a second generation multigene test launched in 2011 [11]. It is a 12 gene reverse transcription-quantitative real-time PCR (RT-qPCR)-based assay, including disease-relevant as well as control and reference genes. It selectively targets the group of ER-positive/HER2-negative breast cancers [11,12,13,14,15,16]. Based on the relative expression of the genes-of-interest a 12-gene molecular score (EP score) is calculated. Subsequently tumour size and lymph node status are taken into account to calculate the clinico-pathological EPclin score, the final test result. The EndoPredict test was validated independently in three large randomized phase III trials with n = 378 (ABCSG-6), n = 1324 (ABCSG-8), and n = 928 (transATAC) patients resulting in a level of evidence of 1 [11,12,13]. In all three validation studies the EndoPredict was an independent predictor of distant recurrence. In the same three cohorts the EndoPredict test was especially investigated for the prediction of early and late distant metastasis and reliably identifies the subgroups of patients with an excellent long-term prognosis after 5 years of only endocrine therapy [17].

The concordance of the test results of the various gene expression assays on the market is currently studied by several groups [18,19,20,21,22,23]. To the best of our knowledge no group has yet compared the MammaPrint and EndoPredict test.

In the present study, we examined the concordance of the risk classification of the MammaPrint and EndoPredict test in 48 ER+/HER-negative breast cancer samples.

## Material and methods

### Patient´s characteristics

48 tissue samples from patients of the Breast Centre Vorarlberg with primary invasive breast cancer which had been analysed with the multi-gene assay MammaPrint during routine patient care between 2007 and 2015 were included in the present study. The median age of the patients was 55 years. The clinico-pathological characteristics of the patients are shown in Table 1. 24 of 48 (50%) of the tumours were invasive ductal carcinomas (IDC), 23/48 (48%) invasive lobular carcinomas (ILC) and 1 tumour (2.1%) was an invasive ductal carcinoma with squamous components. Invasive lobular cancers constitute approximately 15% of all breast cancers. The number of invasive lobular carcinomas in our cohort is obviously higher than that percentage. The decision to conduct the MammaPrint tests (and subsequently the comparative EndoPredict tests) was purely based on the clinical needs. To address this bias the results of the MammaPrint and EndroPredict test were analyzed both for the whole cohort and for ILC and IDC separately. 60% (29/48) of the patients were node-negative and 40% (19/48) node-positive, respectively. The majority of the tumours were positive for ER (96% [46/48]) and PR (81% [39/48]) and negative for HER2 (96% [46/48]). 92% (44/48) of the tumours were positive for ER and negative for HER2 which represent the target group for gene expression profiling and the EndoPredict test as well. The MammaPrint test can be performed on fresh or freshly frozen breast cancer tissue or FFPE tissue [24]. 22 freshly frozen (46%) and 26 FFPE (54%) tumour samples were analysed. MammaPrint test results had been considered together with other clinical and pathological factors by the interdisciplinary tumour board for clinical decision making regarding administration of adjuvant chemotherapy.

**Table 1:** Characteristics of the breast cancer cohort.

Variables	N (%)	Variables	N (%)
**Age**		**Chemotherapy**	
<40	3 (6.3)	Yes	15 (31.2)
>40	45 (93.7)	No	33 (68.8)
			
**Menopause**		**Estrogen receptor**	
premenopausal	18 (37.5)	Positive	46 (95.8)
postmenopausal	26 (54.2)	Negative	2 (4.2)
Unknown	4 (8.3)		
			
**Tumour size**		**Progesterone receptor**	
≤2cm	31 (64.6)	Positive	39 (81.2)
>2cm—≤ 5cm	14 (29.2)	Negative	9 (18.7)
Unknown	3 (6.2)		
			
**Nodal status**		**HER2 status**	
Negative	29 (60.4)	Negative	46 (95.8)
Positive (1–3)	17 (35.4)	Positive	2 (4.2)
Positive (>3)	2 (4.2)		
			
**Grading**			
G1	2 (4.2)		
G2	22 (45.8)		
G3	24 (50)		
			
**Histology**			
IDC/NST	24 (50)		
ILC	23 (47.9)		
IDC with squam. components	1 (2.1)		

The study was approved by the Research Ethics Committee Vorarlberg (No. 2014-4/1).

### Determination of the ER, PR, HER2 and Ki-67 status using immunohistochemistry and dual silver in situ hybridization

The assessment of hormone receptors (ER, PR), HER2 and the cellular proliferation marker Ki-67 was carried out on the assembled tissue microarray representing the areas used for total RNA isolation for EndoPredict testing. The immunohistochemical staining and the Dual silver in situ hybridization (SISH) were performed on the Ventana BenchMark ULTRA (Ventana® Medical Systems Inc., Tucson, USA) using commercial antibodies and DNA probes following manufacturers´ recommendations. For the detection of the primary antibodies for ER, PR and HER2 the BASIC AEC Detection Kit (Ventana®) was used and for Ki-67 the ultraView Universal Alkaline Phosphatase Red Detection Kit (Ventana®). The following primary antibody clones were used: Estrogen receptor (SP1, Ventana®), progesterone receptor (1E2, Ventana®), HER2 (c-erb2, IDLabs, Birmingham, England) and Ki-67 (30–9, Ventana®). All procedures for the SISH analysis were carried out following the recommended protocol of the manufacturer using the INFORM HER2 Dual SISH DNA Probe Cocktail (Ventana®). For the detection of the HER2 probe the ultraView SISH DNP Detection Kit (Ventana®) and for the CEP17 probe the ultraView Red ISH DIG Detection Kit were used, respectively. The cut off for positive ER and PR was set to 1% of positive stained nuclei. HER2 immunohistochemistry was scored as described in the current ASCO guidelines [25]. Ki-67 expression analysis was divided into three categories, in which ≤15% proliferation activity was scored as low proliferation, 16–25% as intermediate and >25% as positive or high proliferation. The used cut-offs were defined in-house. SISH testing was evaluated in reference to the ASCO guidelines. 20 tumour cell nuclei were assessed and the cut-off for positive HER2 status was set as HER2/CEP17 ratio ≥ 2.

### MammaPrint test

Upon the recommendation of the breast cancer tumour board, the samples were sent to the reference laboratory of Agendia (Agendia, Amsterdam, Netherlands) for MammaPrint testing according to their standard operating procedure (SOP) guidelines. For this assay, fresh tissue as well as FFPE tissue can be used [24]. Sections of fresh tissue containing the largest amount of invasive tumour cells were chosen by an experienced pathologist, and sent to Agendia laboratories according to the recommendations of the company. Concerning FFPE material, one representative paraffin block was chosen, containing the largest amount of invasive tumour cells on the corresponding hematoxylin & eosin (H&E) slides. Additionally, 10 unstained serial sections of 5 micrometer thickness per tumour block containing approximately 30% tumour cells were cut from the paraffin blocks, mounted on glass slides and shipped. The test determines the expression of 70 candidate genes and hundreds of reference genes which are measured 6 times each [26]. The multi-gene assay covers the biological motives related to the six hallmarks of cancer including apoptosis, self-sufficiency in growth signals, limitless replicative potential, tissue invasion and metastasis, in-sensitivity to anti-growth signals and sustained angiogenesis [26,27]. By means of this expression profile a risk classification to develop distant metastasis is developed [8,26,28,29,30].

### EndoPredict test

EndoPredict was performed retrospectively using the same archived paraffin blocks assessed by MammaPrint previously. For the 22 fresh tissue samples analyzed with MammaPrint, corresponding FFPE tumour blocks were chosen. First, a tissue section was cut from each sample for H&E staining, the area of invasive tumour cells was identified and marked under the light microscope for tumour cell enrichment and sampling. As recommended by the manufacturer the area of the invasive tumour was at least 30% of the whole tissue section [29]. One adjacent unstained tissue section (thickness of 10μm) was transferred into a 2ml, RNase-free sample tube for total RNA extraction. Total RNA extraction was performed using a manual, silica-coated magnetic bead-based method (Versant® Tissue Preparation Reagent Kit, Siemens Healthcare Diagnostics GmbH, Erlangen, Germany) following the manufacturer´s recommendations. RNA was eluted in 100μl elution buffer. Subsequent quantitative reverse-transcription real-Time PCR (RT-qPCR) analysis was performed to determine the yield of total RNA and to check for DNA contamination. For the determination of the amount of RNA and of residual genomic DNA qPCR assays for the RNA of the 60S ribosomal protein L37a coding gene (RPL37A) as well as for DNA of the β-globin (HBB) gene were used according to manufacturer’s instructions. If needed, an additional DNase digestion was performed to recover genomic DNA-free total RNA.

The multigene assay EndoPredict (Sividon Diagnostics GmbH, Cologne, Germany / Myriad Genetics, Zurich, Switzerland) uses the quantitative reverse-transcription real-Time PCR (RT-qPCR) technique to detect the expression of eight genes-of-interest (AZGP1, BIRC5, DHCR7, IL6ST, MGP, RBBP8, STC2, UBE2C) and three reference genes (CALM2, OAZ1, RPL37A) applied for normalization as well as of the amount of residual genomic DNA (HBB) using the SuperScript III Platinum One-Step Quantitative RT-PCR Kit with ROX (Invitrogen, Carlsbad, USA) in a VERSANT® kPCR System (Siemens Healthcare Diagnostics GmbH)[14,15]. The corresponding sequences of primer and FAM/TAMRA-labelled probes were published previously [11]. After upload of PCR data, tumour size and nodal status the EP and EPclin scores were calculated with the web-based EndoPredict Report Generator. The EP score is purely based on the gene-expression data. The EPclin score, the final result of the EndoPredict test which is used for clinical decision making, considers tumour size and nodal status in addition to the molecular EP score [11]. Using predefined cut off values patients were classified by the software into low or high risk of distant metastasis. The cut off value for the EP score was 5.0 and for the EPclin score 3.32867 [11].

For all 48 study samples the extraction of a sufficient amount of total RNA and generation of valid results was possible. Scores and risk groups were generated for each patient and compared to the risk classification of the MammaPrint test. The analyses for the EndoPredict test were carried out blinded to the outcome data and the MammaPrint results in the lab of the manufacturer Sividon Diagnostics in Germany. All reagents for the EndoPredict analysis were provided by Sividon Diagnostics. In addition to that the author(s) received no specific funding for this work. Sividon Diagnostics had no role in study design, data collection and analysis, decision to publish, or preparation of the manuscript. This does not alter our adherence to PLOS ONE policies on sharing data and materials.

### Interdisciplinary tumour board

An interdisciplinary tumour board is conducted weekly at the Breast Center Vorarlberg for the management of breast cancer patients including medical oncologists, surgeons, radiation therapists, pathologists, radiologists, and other specialists for treatment planning. The results of the different departments are assembled and interpreted. Since 2007 the results of the gene expression profiling test MammaPrint are taken into account additionally. In the framework of a retrospective and virtual tumour board the results of the MammaPrint risk classifications were replaced by the EndoPredict test results. During a decision impact analysis the identity of the patients and treatment recommendations were blinded and reassessed taking EndoPredict risk classifications into account.

### Statistics

All statistical analyses were performed using R version 3.0.2 [31]. Correlation between continuous variables was evaluated using Pearson´s correlation coefficient (*r*) or Spearman´s rank correlation coefficient (*ρ*) using *cor*.*test()* function. In case of categorical values contingency tables were generated. Concordance was then calculated as the number of matching cases divided by the total number of observations and evaluated using Cohen´s kappa (*κ*), calculated as

$$\kappa =\frac{p_{o}-p_{c}}{1-p_{c}},$$

with p_o_ being the observed concordance and p_c_ the proportion of cases for which agreement is expected by chance. Additionally, two-sided Fisher´s exact test as implemented in the *fisher*.*test()* function was used to evaluate the observed concordance. For all statistical tests a level of significance of 95% was applied.

## Results

### MammaPrint test results

A valid risk classification by the MammaPrint test was possible in 45 of the 48 cases (94%). In 3 of 48 tumours (6%) the RNA quality was too low for a proper risk assessment. 17 of these 45 tumours (38%) were classified as low risk and 28 (62%) as high risk. When comparing the tumour grade with the risk classification we found no statistically significant correlation with the MammaPrint test results (p = 0.413, S1 Fig).

### EndoPredict test results

The EndoPredict test provided valid test results for all 48 tumours including an 12-gene molecular EP score and EPclin risk score. For statistical analysis 4 of 48 (8%) of the patients had to be excluded, because their tumours were estrogen receptor negative or HER2 positive and did not meet the inclusion criteria of the EndoPredict test (S1 Fig).

According to the molecular EP score, 18% (8/44) of the patients were classified as low risk and 82% (36/44) of the patients as high risk. The EPclin score re-classified 20% (9/36) EP high risk patients into the low risk group resulting in 39% (17/44) of the patients with a low risk classification and in 61% (27/44) of the patients with a high risk of disease recurrence. A strong concordance of 80% was detected between the EP score and the EPclin score-based risk classifications (κ = 0.522, p = 0.137 × 10^−3^). This was to be expected since the EPclin score is directly depending on EP score. When comparing the tumour grade with the risk classification we found no statistically significant correlation with the EP score (p = 0.184) and the EPclin score (p = 0.062), respectively.

### MammaPrint versus EP Score-based risk classification

**Fig 1:**
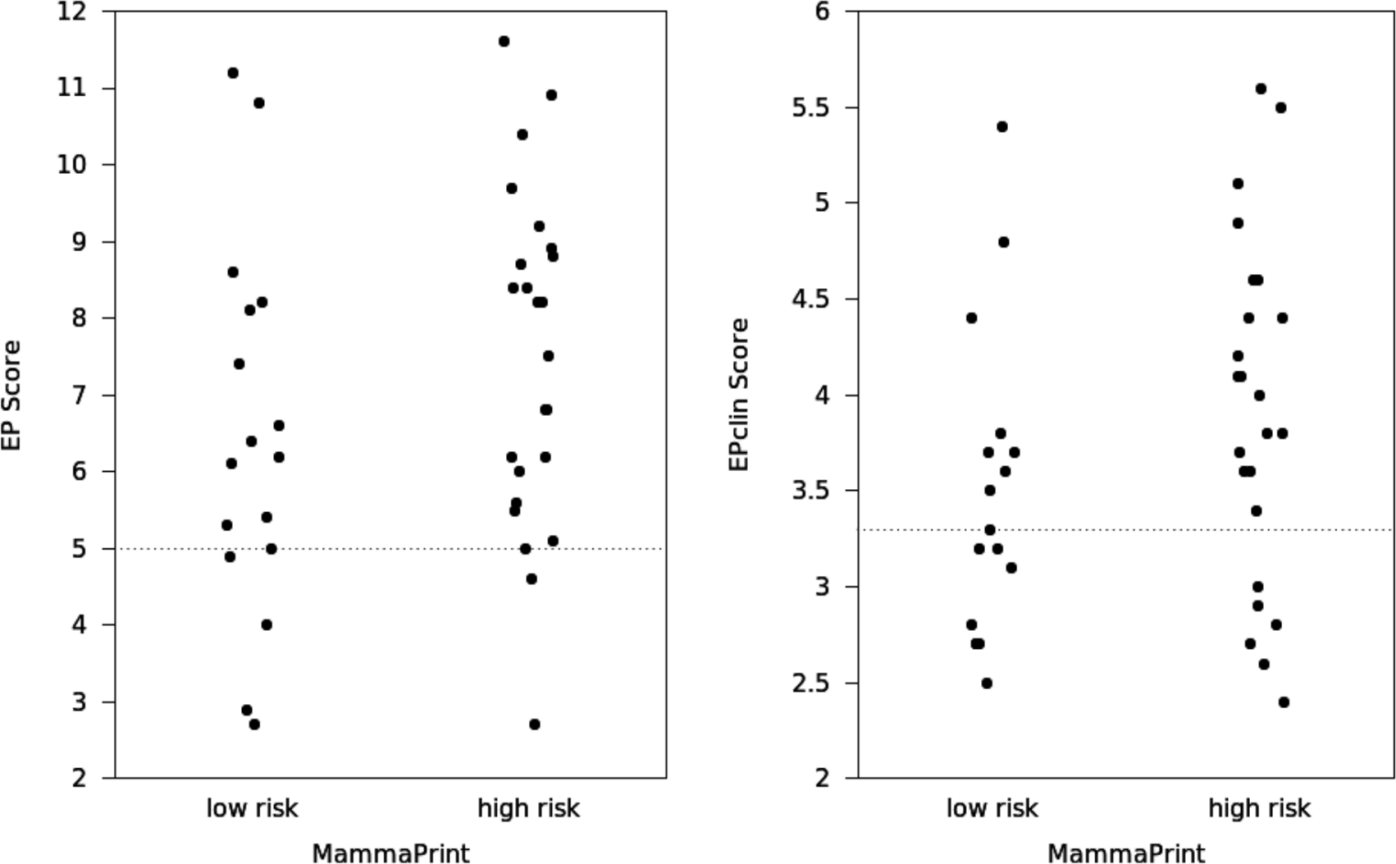
Correlation between the MammaPrint risk classification and the EP score (left) and EPclin score (right). Threshold values for EP- and EPclin-based risk classification are indicated as dashed lines.

Comparing the MammaPrint and EP score-based risk classifications, an overall concordance of 63.4% (26/41) was found. Both tests classified 10% (4/41) and 54% (22/41) of the comparable cases as low risk and high risk, respectively. However, 32% (13/41) of the cases classified as low risk by MammaPrint were classified as high risk by the EP score. 5% (2/41) were classified as high risk by the MammaPrint and low risk by the EP score. As reflected by Cohen´s Kappa coefficient κ = 0.168, there was only a week agreement between the two molecular classifications (Fig 1; Table 2). Fisher’s exact test did not confirm a statistically significant agreement between the two classification methods beyond of what would have been expected by chance (p = 0.212). Separate analysis of ILC and IDC revealed a tendency of a better correlation of the MammaPrint and EP score in ILC. However, this was not statistically significant (Fisher´s exact test) (S1 and S2 Tables).

**Table 2:** Correlation between EP/ EPclin score and MammaPrint.

n = 41		MP low risk	MP high risk	Overall concordance	Cohen´s κ	Fisher´s Exact test
**∑**	EP low risk	4 (9.8%)	2 (4.9%)			
	EP high risk	13 (31.7%)	22 (53.7%)	63.4	0.168	0.212
**G1+2**	EP low risk	3 (15%)	1 (5%)			
	EP high risk	7 (35%)	9 (45%)	60.0	0.200	0.582
**G3**	EP low risk	1 (4.8%)	1 (4.8%)			
	EP high risk	6 (28.5%)	13 (61.9%)	66.7	0.087	1
**∑**	EPclin low risk	9 (22.0%)	6 (14.6%)			
	EPclin high risk	8 (19.5%)	18 (43.9%)	65.9	0.284	0.102
**G1+2**	EPclin low risk	6 (30%)	4 (20%)			
	EPclin high risk	4 (20%)	6 (30%)	60.0	0.200	0.656
**G3**	EPclin low risk	3 (14.3%)	2 (9.5%)			
	EPclin high risk	4 (19.0%)	12 (57.1%)	71.4	0.308	0.280

∑: whole cohort; G1+2: only tumours of grade 1+2: G3: only tumours of grade 3.

### MammaPrint versus EPclin Score-based risk classification

Only a poor and non-significant overall concordance of 66% (27/41) was found between MammaPrint and EPclin score-based risk classifications (κ = 0.284, p = 0.102) (Fig 1; Table 2). For 22% (9/41) of the patients both tests classified the tumours as low risk and for 44% (18/41) as high risk. However, of the 17 MammaPrint low risk cases, only 9 were low risk based on the EPclin score (53% agreement). A better agreement was seen in the high risk group. Of the 24 MammaPrint high risk cases 18 were also high risk based on the EPclin score (75% agreement). Separate analysis of the ILC and IDC subsets showed a tendency of a better correlation of the MammaPrint and EPclin test result in ILC than in IDC. Discrepancies occurred in 4 of 20 cases of ILC and in 10 of 21 of IDC. These differences were however, not statistically significant (Fisher´s exact test). (S1 and S2 Tables).

### Correlation of the Ki-67 index with the MammaPrint, the EP and EPclin score

**Fig 2:**
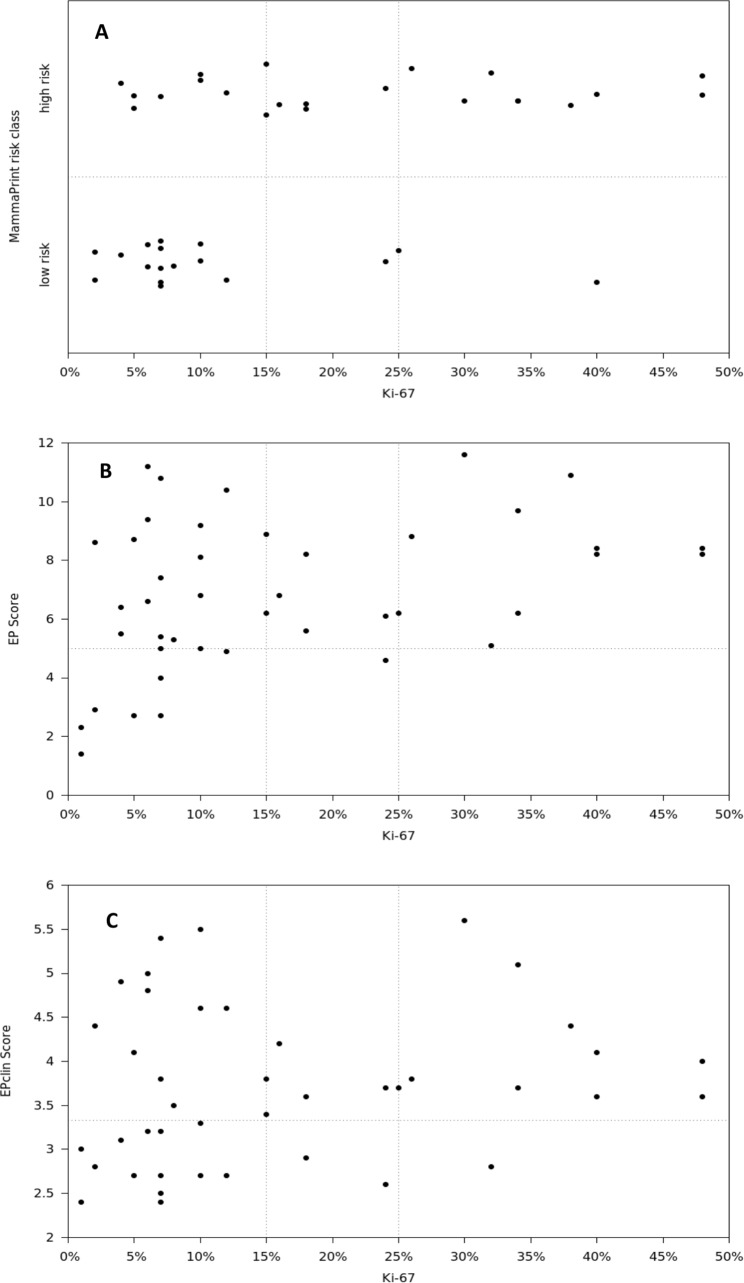
Correlation of the Ki-67 proliferation indices and MammaPrint (A), EP (B) or EPclin (C) score risk classification (threshold values of Ki-67 are registered as vertical dashed lines. Tumour cells with a Ki-67 proliferation index <15% were scored as negative, 16%-25% as intermediate and >25% as positive).

When comparing the Ki-67 proliferation indices with the molecular risk classifiers we found a statistically significant correlation with the MammaPrint test results (p = 0.499 × 10^−2^ using Fisher’s Exact test; Fig 2A) and the molecular EP scores, (p = 0.241 x 10^−2^, r = 0.44; Fig 2B and S1 Table). By contrast, there was no significant relation between the Ki-67 index and the EPclin score (p = 0.099, r = 0.25; Fig 2C and S1 Table).

### Decision impact analysis

In the framework of interdisciplinary tumour board meetings the effect of the MammaPrint and EndoPredict test results on the choice of therapy were compared.

**Fig 3:**
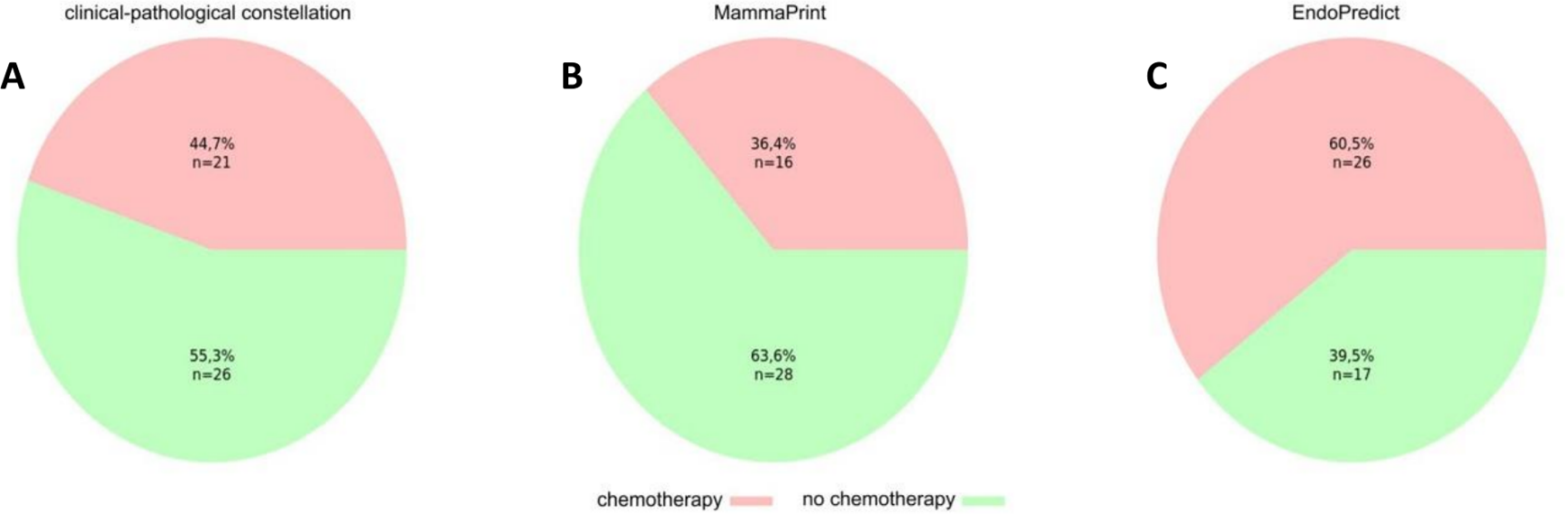
Decision impact analysis. A) Initial choice of therapy based on taking only the clinico-pathological parameters tumour size, nodal status and hormone receptor status as well as HER2 status into account B) Actual choice of therapy based on the inclusion of the MammaPrint test result C) Hypothetical choice of therapy based on the inclusion of the EPclin Score test result instead of the MammaPrint results.

During the interdisciplinary tumour boards of the Breast Centre Vorarlberg in the years 2007–2015, in an initial step the choice of therapy was considered, taking only classical clinico-pathological parameters into account. These included tumour size, nodal status, hormone receptor and HER2 status and the Ki-67 proliferation index. Sufficient information was available in 47 patients. Based on these data for 45% (21/47) of the patients an adjuvant chemotherapy would have been the choice of therapy, whereas in 55% (26/47) the clinicians would have refrained from chemotherapy recommendation (Fig 3A). Thereafter, the MammaPrint test results were included leading to the actual therapeutic strategy chosen for each patient. MammaPrint results were available for 44 patients and led to a reduction of patients regarded to need adjuvant chemotherapy. Thus, only 36% (16/44) of the patients actually received chemotherapy (Fig 3B). In tumour board meetings of the year 2015, all historic patients were presented again and the MammaPrint test results were replaced by the EndoPredict test results as a hypothetical construct. In this context the recommendation for chemotherapy would have increased to 61% (26/44) of the patients (Fig 3C). 9% (4/44) of the patients were excluded because of a negative hormone receptor (ER negative) or positive HER2 status.

### Follow up data

In the framework of this study follow up data of all patients and in 69% (33/48) risk classifications of both gene expression profiling tests (MammaPrint and EndoPredict) were available. 4/33 (12.1%) patients developed distant metastasis during median observation time of 53 months (range 20–96 months). The patients developed distant metastasis after 25, 27, 29 and 31 months, respectively (Table 3). The primary tumours of all of these 4 patients were classified as high risk tumours by the EndoPredict test. The MammaPrint classified 1 of the 4 metastasizing tumours as low risk.

**Table 3:** Overview of the risk classification of patients with distant metastasis during observation time.

Sample ID	Metastasis (Months)	MammaPrint	EP score	EP score value	EPclin score	EPclin score value	Ki-67 (%)	Recurrence site
**17**	25	high risk	high risk	11.4	high risk	4.5	40	Brain^1^
**28**	27	low risk	high risk	11.2	high risk	4.8	24	Bone^2^
**38**	29	high risk	high risk	10.9	high risk	4.4	60	Bone^3^
**44**	31	high risk	high risk	9.7	high risk	5.1	34	Bone^3^
								

^1^ chemotherapy recommended but refused by patient

^2^ chemotherapy omitted based on clinical parameters and MammaPrint test result

^3^ chemotherapy recommended and applied

## Discussion

Treatment decisions based on clinico-pathological factors only result in overtreatment of a significant fraction of early breast cancer patients. It has been estimated that approximately 85% of the patients do not benefit from adjuvant chemotherapy regarding 10-year breast cancer-specific survival [2,3,4,6]. This dilemma led to the introduction of various diagnostic multigene expression assays such as MammaPrint, Oncotype DX, Breast Cancer Index, PAM50 and the EndoPredict test. However, the value of these tests to predict prognosis and individualize adjuvant chemotherapy is a matter of an ongoing debate and only a few studies have attempted to compare these assays in a head to head fashion [18,20,21,22,32,33,34,35,36,37,38,39].

To our knowledge, the present study is the first to compare the first generation test MammaPrint and the second generation test EndoPredict. We found a discrepant risk classification in 14 of the 41 (34%) directly comparable tumours which would have resulted in a different treatment in 38% of our patients. Compared to the MammaPrint the EndoPredict test results would have led to significantly higher percentage of patients put on adjuvant chemotherapy (61% instead of 36%). Our results are provocative and support previous observations that multigene expression assay are neither easily interchangeable nor comparable [21,22,34].

MammaPrint was one of the first gene expression assays on the market. It is a 70-gene classifier and was developed to distinguish patients with a high probability of metastasis free survival from patients with high risk to develop distant metastases within 5 years of diagnosis [8,30]. The genes analysed are related to various characteristics of cancer cells including self-sufficiency in growth signals, insensitivity to anti-growth signals, deregulation of apoptosis, limitless replication, tissue invasion and metastasis, and sustained angiogenesis [26]. The test was analysed in various validation studies and proved to be of prognostic value independent of tumour stage and histopathologic factors such as tumour grade and the Nottingham prognostic index [7,40,41,42,43]. A common observation of these studies was that the MammaPrint works better in the prognostication of early recurrences. In a meta-analysis it was also shown that the test may also provide predictive information when applying chemotherapy in high risk patients [44]. The potential benefit of the MammaPrint test was recently highlighted in the phase-3 study of 6993 early-stage breast cancer patients (MINDACT). In this comprehensive study it was shown that 46% of women with a high clinical but low genetic risk do not require chemotherapy. Additionally, it was demonstrated that the 5-year overall survival of patients with estrogen-positive, HER2-positive or negative and either node-negative or node-positive breast cancer was similar [10]. A re-evaluation of the patients after an extended time period of 10 years will be of great interest.

However, recently so called second generation assays have entered the market. They address the main target group of estrogen receptor positive tumours more specifically and are thought to predict not only early but also late recurrences within a time frame of 10 years. Furthermore, they do also incorporate classical clinico-pathologic risk factors [22,34]. The first of the second generation tests launched is the EndoPredict assay. It was introduced as a novel multigene classifier to assess the prognosis in ER+/HER2-negative breast cancer patients. It is a 12 gene expression test including genes related to proliferation and to hormone receptor activity [11]. In contrast to the MammaPrint, EndoPredict test is designed to be performed decentralized which—by many pathologists—is regarded a major advantage [14]. The robustness of the EndoPredict assay in a decentralized test setting using formalin-fixed, paraffin-embedded tissue (FFPE) was successfully proven in seven European pathology institutions with 100% concordance of test results between the different sites [14]. The EndoPredict was validated in three phase III trials involving patients on endocrine therapy only (ABCSG6, ABCSG8, transATAC) and proved to be a significant risk factor classifier for distant disease recurrence [12;45]. A remarkable result of these studies was that approximately 50% of the patients were determined to be “high risk” and would have been subjected to treatment with chemotherapy, if the molecular EP score would have been taken as the basis for therapeutic decision [11,19,23]. A similar, but even more pronounced observation was made in the present study. More than 80% of our patients were classified as high risk by the EP score. This might reflect a selection bias since a relatively high percentage (50%) of our patients presented with grade 3 tumours based on histopathologic grading. However, the final test result of the EndoPredict test combines the molecular EP score with tumour size and nodal status. Based on the resulting EPclin scores, 20% (9/36) of our EP high risk patients were re-classified into the low risk group resulting in 61% (27/44) of our patients with a high risk of disease recurrence. A similar observation of a “risk-reduction” by the inclusion of traditional clinical parameters was made in the above-mentioned clinical validation trials of EndoPredict. Overall, the percentage of high risk patients in our patient group seemed to correlate when comparing the EPclin score and the MammaPrint. However, of the 24 MammaPrint high risk cases only 18 were high risk by EPclin score resulting in only a weak correlation (75% agreement). Similarly, of the 17 MammaPrint low risk cases, only 9 were low risk by EPclin score (53% agreement). Thus, discrepancies between the EPclin score and the MammaPrint occurred in more than a third of our patients (14 of 41 patients). Based on the purely clinical decision to conduct gene expression tests in our cohort the percentage of lobular cancers was higher than expected. Interestingly, there was a tendency of a better correlation between the MammaPrint and EPclin test result in ILC than in IDC. However, these differences were not statistically significant.

The reason for a higher proportion of EPclin high risk patients in our cohort compared to most of the previous reports might be a large proportion of patients with node positive disease (40%) and/or grade 3 tumours (50%) reflecting a clinically higher risk cohort.

A similar correlation of only 75% was described between the EndoPredict test and the Oncotype Dx Recurrence Scores. [20]. The Oncotype Dx is a RT-qPCR based 21-gene assay, including 16 genes related to proliferation and 5 housekeeping-genes for normalization [46,47].

Recently, the EPclin score was directly compared to purely clinico-pathologic risk classifications and was found to be superior to these classifiers [17]. Furthermore, the EPclin score was used in a recent decision impact study and led to a change of therapy in 37.7% of patients with 25.4% patients placed on endocrine therapy alone and 12.3% on additional chemotherapy [19]. Compared to the clinico-pathologic parameters assessed for our study group the application of the EPclin score would have placed an additional 15.8% on adjuvant chemotherapy, which can be explained by the high risk cohort (40% node positive; 50% grade 3 tumours). By contrast, the MammaPrint led to a reduction of chemotherapies by 8.3% in our study.

Due to the low number of samples, short follow-up data and the low event rate an assessment of the diagnostic potential of both gene expression assays was not possible. Only descriptive data analysis was performed. Our study cannot claim which gene expression assay gave the more accurate risk assessment but raises the important question if all of the patients received the right treatment. 4 patients developed distant metastasis during observation time. EndoPredict identified all of them as high risk while the MammaPrint misclassified one of the metastasizing tumours as low risk. In this case chemotherapy was omitted based on clinical parameters and the MammaPrint test result. In total our study underlines the necessity of further comparative studies of the currently used gene expression assays.

## Supporting information

S1 Table Statistical evaluation of the correlation between EP- /EPclin score and MammaPrint in the ILC subset. ∑: ILC subset; G1+2: only tumours of grade 1+2: G3: only tumours of grade 3.
(DOCX)

S2 Table Statistical evaluation of the correlation between EP- /EPclin score and MammaPrint in the IDC subset. ∑: IDC; G1+2: only tumours of grade 1+2: G3: only tumours of grade 3.
(DOCX)

S3 Table Concordance of the Ki-67 proliferation indices and risk classification of the gene expression assays. (DOCX)

S1 Fig CONSORT diagram of the study cohort. (TIF)

## Abbreviations

ABCSG: Austrian Breast & Colorectal Cancer Study Group
AEC: 3-amino-9-ethylcarbazole
ASCO: American Society of Clinical Oncology
AZGP1: Alpha-2-glycoprotein-1, zinc-binding
BIRC5: Baculovirus IAPrepeat-containing 5
CALM2: Calmodulin 2
CEP17: centromere enumerator probe for chromosome 17
Ct: cycle threshold
DHCR7: 7-Dehydrocholesterol reductase
DIG: digoxigenin
DNA: deoxyribonucleic acid
ER: estrogen receptor
FFPE: formalin-fixed, paraffin-embedded (tissue)
H&E: hematoxylin and eosin
HBB: hemoglobin, beta
HER2: human epidermal growth factor receptor 2
IDC: invasive ductal carcinoma
IHC: immunohistochemistry
IL6ST: interleukin 6 signal transducer
ILC: invasive lobular cancer
MGP: Matrix GLA protein
NST: no special type
OAZ1: Ornithine decarboxylase antizyme 1
PR: progesterone receptor
RBBP8: Retinoblastoma binding protein 8
RNA: ribonucleic acid
ROX: 6-Carboxyl-X-Rhodamine
RPL37A: Ribosomal protein L37a
RT-qPCR: quantitative reverse transcription polymerase chain reaction
SISH: silver in situ hybridization
STC2: Stanniocalcin 2
UBE2C: Ubiquitin-conjugating enzyme E2C
