# Supplementary material for: MammaPrint versus EndoPredict: Poor correlation in disease recurrence risk classification of hormone receptor positive breast cancer
Source: PLoS One. 2017 Aug 29;12(8):e0183458. doi: 10.1371/journal.pone.0183458 (PMC5574574; doi:10.1371/journal.pone.0183458)
Supplement: S1 Table — ∑: ILC subset; G1+2: only tumours of grade 1+2: G3: only tumours of grade 3. (DOCX) [file pone.0183458.s001.docx]

**Table S1: Statistical evaluation of the correlation between EP- /EPclin score and MammaPrint in the ILC subset**

| n=20 |  | MP low risk | MP high risk | Overall  concordance | Cohen´s  κ | Fisher´s  Exact test |
| --- | --- | --- | --- | --- | --- | --- |
| ∑ | EP low risk | 2 (10.0%) | 0 (0.0%) |  |  |  |
|  | EP high risk | 6 (30.0%) | 12 (60.0%) | 70.0 | 0.286 | 0.147 |
| G1+2 | EP low risk | 2 (25.0%) | 0 (0.0%) |  |  |  |
|  | EP high risk | 2 (25.0%) | 4 (50.0%) | 75.0 | 0.500 | 0.427 |
| G3 | EP low risk | 0 (0.0%) | 0 (0.0%) |  |  |  |
|  | EP high risk | 4 (33.3%) | 8 (66.6%) | 66.7 | 0.000 | 1 |
| ∑ | EPclin low risk | 5 (25.0%) | 1 (5.0%) |  |  |  |
|  | EPclin high risk | 3 (15.0%) | 11 (55.0%) | 80.0 | 0.565 | 0.018 |
| G1+2 | EPclin low risk | 3 (37.5%) | 0 (0.0%) |  |  |  |
|  | EPclin high risk | 1 (12.5%) | 4 (50.0%) | 87.5 | 0.750 | 0.143 |
| G3 | EPclin low risk | 2 (16.7%) | 1 (8.3%) |  |  |  |
|  | EPclin high risk | 2 (16.7%) | 7 (58.3%) | 75.0 | 0.400 | 0.236 |
