# Supplementary material for: MammaPrint versus EndoPredict: Poor correlation in disease recurrence risk classification of hormone receptor positive breast cancer
Source: PLoS One. 2017 Aug 29;12(8):e0183458. doi: 10.1371/journal.pone.0183458 (PMC5574574; doi:10.1371/journal.pone.0183458)
Supplement: S2 Table — ∑: IDC; G1+2: only tumours of grade 1+2: G3: only tumours of grade 3. (DOCX) [file pone.0183458.s002.docx]

**Table S2: Statistical evaluation of the correlation between EP- /EPclin score and MammaPrint in the IDC subset**

| n=21 |  | MP low risk | MP high risk | Overall  concordance | Cohen´s  κ | Fisher´s  Exact test |
| --- | --- | --- | --- | --- | --- | --- |
| ∑ | EP low risk | 2 (9.5%) | 2 (9.5%) |  |  |  |
|  | EP high risk | 7 (33.3%) | 10 (47.6%) | 57.1 | 0.060 | 1 |
| G1+2 | EP low risk | 1 (8.3%) | 1 (8.3%) |  |  |  |
|  | EP high risk | 5 (41.7%) | 5 (41.7%) | 50.0 | 0.000 | 1 |
| G3 | EP low risk | 1 (11.1%) | 1 (11.1%) |  |  |  |
|  | EP high risk | 2 (22.2%) | 5 (55.6%) | 66.7 | 0.182 | 1 |
| ∑ | EPclin low risk | 4 (19.0%) | 5 (23.8%) |  |  |  |
|  | EPclin high risk | 5 (23.8%) | 7 (33.3%) | 52.4 | 0.028 | 1 |
| G1+2 | EPclin low risk | 3 (25.0%) | 4 (33.3%) |  |  |  |
|  | EPclin high risk | 3 (25.0%) | 2 (16.7%) | 41.7 | -0.167 | 1 |
| G3 | EPclin low risk | 1 (11.1%) | 1 (11.1%) |  |  |  |
|  | EPclin high risk | 2 (22.2%) | 5 (55.6%) | 66.7 | 0.182 | 1 |
