# Supplementary material for: MammaPrint versus EndoPredict: Poor correlation in disease recurrence risk classification of hormone receptor positive breast cancer
Source: PLoS One. 2017 Aug 29;12(8):e0183458. doi: 10.1371/journal.pone.0183458 (PMC5574574; doi:10.1371/journal.pone.0183458)
Supplement: S3 Table — (DOCX) [file pone.0183458.s003.docx]

Table S3: Concordance of the Ki-67 proliferation indices and risk classification of the gene expression assays.

|  | | MammaPrint | | EP-Score | | EPclin-Score | |
| --- | --- | --- | --- | --- | --- | --- | --- |
|  |  | low risk | high risk | low risk | high risk | low risk | high risk |
| Ki-67 | low | 35.9% (14/39) | 17.9% (7/39) | 16.7% (7/42) | 40.4% (17/42) | 30.9% (13/42) | 26.2% (11/42) |
|  | intermediate | 5.1% (2/39) | 15.4% (6/39) | 2.4% (1/42) | 16.7% (7/42) | 4.8% (2/42) | 14.3% (6/42) |
|  | positive | 2.6% (1/39) | 23.1% (9/39) | 0% (0/42) | 23.8% (10/42) | 2.4% (1/42) | 21.4% (9/42) |

⨯ T
