# Supplementary figures and images for: MammaPrint versus EndoPredict: Poor correlation in disease recurrence risk classification of hormone receptor positive breast cancer
Source: PLoS One. 2017 Aug 29;12(8):e0183458. doi: 10.1371/journal.pone.0183458 (PMC5574574; doi:10.1371/journal.pone.0183458)

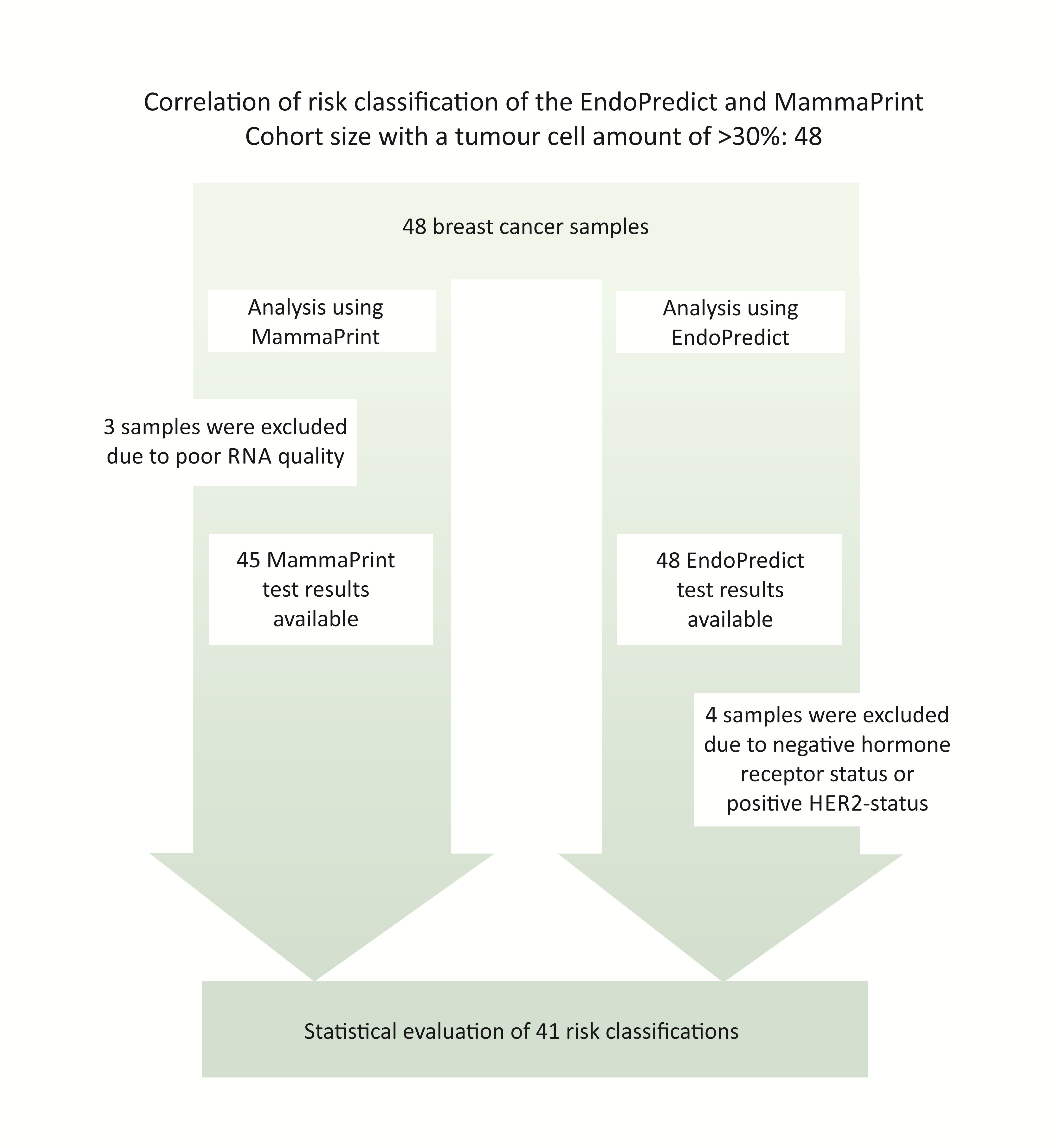

Supplement: S1 Fig — (TIF) [file pone.0183458.s004.tif]
